# A novel “total pituitary hormone index” as an indicator of postoperative pituitary function in patients undergoing resection of pituitary adenomas

**DOI:** 10.18632/oncotarget.15978

**Published:** 2017-03-07

**Authors:** Shousen Wang, Biao Li, Chenyu Ding, Deyong Xiao, Liangfeng Wei

**Affiliations:** ^1^ Department of Neurosurgery, Fuzhou General Hospital, Fujian Medical University, Fuzhou, P. R. China

**Keywords:** hypopituitarism, non-functioning pituitary adenoma, pituitary adenoma, prolactinoma, surgery

## Abstract

The purpose of this study was to investigate the differences between pre- and postoperative pituitary hormone levels in patients undergoing surgical resection of pituitary adenoma and to identify factors associated with preoperative hypopituitarism.

Data from 81 patients with histologically confirmed functioning and non-functioning pituitary adenomas (NFPA) who underwent transsphenoidal resection from January 2011 to December 2013 were retrospectively analyzed. Logistic regression was applied to analyze factors associated with preoperative hypopituitarism. In patients with functioning pituitary adenomas, GH and PRL levels declined after the operation; TSH, FSH and LH levels returned to preoperative values after an initial decline at postoperative day 1. In contrast, with the exception of a postoperative reduction in PRL level, NFPA patients had postoperative ACTH, TSH, FSH and LH levels at 4 months follow-up that were similar to preoperative levels. Similarly, a decrease in total hormone index was observed following surgery irrespective of NFPA type and in null-cell type NFPA patients with values increasing over the 4-month follow-up period. A higher percentage of patients receiving partial resection had high PRL levels (≥200 ng/ mL) compared to those receiving complete resection. Age (*P* = 0.041) and male sex (*P* = 0.004) were significantly associated with preoperative hypopituitarism. In conclusion, the postoperative total hormone index decreased immediately following surgery in all patients with pituitary adenoma who underwent resection, and then increased over the follow-up period. The extent of surgical resection correlated with PRL levels >200 ng/mL. Age and male sex were also independent risk factors for preoperative hypopituitarism.

## INTRODUCTION

Pituitary adenomas are benign tumors that account for 10-15% of all intracranial tumors [1]. Some pituitary adenomas are classified as non-functioning pituitary adenomas (NFPAs), including gonadotropin-cell adenomas, null cell adenomas and silent adenomas [2]. NFPAs do not secrete hormones, or secrete levels such that they are undetectable in serum and without clinical symptoms [3]. Clinically “silent” NFPA may result in supra-normal serum hormone concentrations of the specific adenoma cell type but without the usual clinical manifestations expected with excessive levels of that hormone [4]. Partial or complete hypopituitarism may occur in NFPA patients when 95% of pituitary cells are damaged [2, 5]. In contrast, functional pituitary adenomas (i.e., growth hormone [GH], adrenocorticotropic hormone [ACTH], thyroid stimulating hormone [TSH], and prolactin [PRL] adenomas or prolactinomas) secrete sufficient hormones to cause corresponding signs and symptoms of hyperprolactinemia, acromegaly, or Cushing’s syndrome. Symptoms of larger adenomas (macroadenomas) are due to compression of peripheral cells, resulting in insufficient secretion of one or more hormones [6], visual loss and headache.

Transsphenoidal surgery is a leading treatment for pituitary adenomas, achieving long-term cure or control in 70-80% of cases in select tertiary centers [7-10]. Furthermore, this approach is associated with less postoperative reduction in pituitary function in patients with NFPAs [11] with normalization of most symptoms within 3 months [12]. The main advantages of surgical treatment include the rapid relief of symptoms caused by excessive hormone secretion and prevention of permanent damage to target organs [7-9]. Potential improvement in pituitary function is an important goal of surgery, and a meta-analysis revealed that remission rates after transsphenoidal surgery are 50-70% for macroadenomas and 80-90% for microadenomas [8]. However, Vargas et al. [10] reported that endocrine deficiencies do not always resolve in NFPA patients undergoing surgery, and the persistence of oculomotor abnormalities after surgery is similar to that observed with conservative treatment. Kristof et al. [13] found that remission rates were significantly higher and recurrence lower in patients with GH- and ACTH-secreting adenomas as compared to PRL-secreting adenomas.

Differences are expected between preoperative and postoperative pituitary hormone secretion in patients with pituitary adenomas. Obvious reductions in serum hormone levels within one week after surgery compared to levels at one month and 3-6 months follow-up have been observed, suggesting that hormone levels measured within one week after surgery may reflect the extent of surgical resection and short-term outcomes [13]. Conversely, recovery of pituitary function following surgical resection of NFPAs may also occur [14]. Although up to 90% of patients with NFPA and 10%-50% of those with functioning adenomas have preoperative functional deficits [15], few studies have evaluated differences in total pituitary function before and after surgical resection as a way to evaluate preoperative hypopituitarism. Instead, studies have focused on changes in specific hormones. Therefore, we hypothesized that longitudinal analysis of pituitary hormones at different time points before and after surgery may reveal preoperative and postoperative “total” hormone levels, which may facilitate the evaluation of the impact of surgery on managing pre-surgical hypopituitarism. We have designed a novel “total hormone index”, and the present study aimed to evaluate differences between pre- and postoperative pituitary hormones and related time trends after pituitary adenoma resection based on the proposed “total hormone index” as well as individual hormone levels. A secondary objective of the study was to identify factors associated with preoperative hypopituitarism.

## RESULTS

Data of 81 patients aged 19-75 years were included in analysis. Patients’ baseline characteristics are summarized in Table 1. Among all patients, 53.1% were males, 58% had NFPA adenoma, 58% received complete tumor resection, 42% had cystic degeneration, and 25.9% had null-cell adenoma.

**Table 1:** Baseline characteristics of 81 patients

	mean ± standard deviation / n (%)
Age, years	44.8 ± 12.3
Male gender	43(53.1)
Type of adenoma	
Gonadotropin	18 (22.2)
Prolactin	18 (22.2)
Null-cell	21 (25.9)
Others^1^	24 (29.6)
NFPA adenoma	47 (58.0)
GnH	18 (38.3)
PRL	2 (4.3)
Others	6 (12.8)
Null cell	21 (44.7)
Maximum diameter of pituitary adenoma, mm	2.3 ± 0.8
< 10	4 (4.9)
10-40	72 (88.9)
≥ 40	5 (6.2)
Tumor volume, cm^3^	61.8 ± 59.3
Level of tumor resection	
Complete	47 (58.0)
Massive	23 (28.4)
Partial	11 (13.6)
Grade of cavernous sinus invasion	2.1 ± 0.9
Grade of suprasellar extension	2.5 ± 1.0
Total score of invasion	6.5 ± 2.1
Cystic degeneration	34 (42.0)

Abbreviations: NFPA: non-functioning pituitary adenomas

^1^ Other types of adenoma include growth hormone (GH), adrenocorticotropic hormone ACTH), thyroid-stimulating hormone (TSH), and plurihormonal pituitary type

### Differences and time trends between pre- and postoperative levels of 6 pituitary hormones and total hormone index by NFPA type

As shown in Table 2, the time trends of pituitary hormones varied by the presence of NFPA. In patients without NFPA, the levels of GH, TSH, PRL, FSH, and LH significantly changed over time (*P*≤0.006); however, ACTH levels appeared stable throughout the study period. At day 1 following surgery, significant differences were found between mean pre- and postoperative levels of TSH (from 1.5 ng/mL to 0.8 ng/mL), PRL (from 459.4 ng/mL to 183.0 ng/mL), and LH (from 3.2 ng/mL to 2.2 ng/mL). However, with the exception of PRL, the levels increased to preoperative values at 4 months follow-up. The GH level in patients without NFPA was significantly reduced at 4 months after the operation from 6.8 to 1.7 ng/mL, and the reduction in PRL level in patients without NFPA remained significant at 4 months (*P*<0.001; Table 2).

**Table 2:** Differences between pre- and postoperative pituitary hormone levels in all patients^1^

Hormone	NFPA	Preoperative	Postoperative Day 1	Postoperative Day 7	Postoperative Month 4	*P*
GH, ng/mL	No (n=34)	6.8 ± 2.0 (1 ± 0)	5.1 ± 2.0 (0.75 ± 0.01)	2.5 ± 0.9 (0.37 ± 0)	1.7 ± 0.6 (0.25 ± 0)^**a**^	**<0.001**
	Yes (n=47)	1.2 ± 0.4 (1 ± 0)	0.7 ± 0.1 (0.58 ± 0.01)	0.6 ± 0.2 (0.50 ± 0)	0.8 ± 0.2 (0.67 ± 0.01)	0.314
ACTH, ng/mL	No (n=34)	23.0 ± 5.0 (1 ± 0)	17.9 ± 4.2 (0.78 ± 0.01)	17.6 ± 2.1 (0.77 ± 0.01)	23.5 ± 2.8 (1.02 ± 0.01)	0.138
	Yes (n=47)	21.0 ± 2.7 (1 ± 0)	15.9 ± 3.6 (0.76 ± 0.01)^**a**^	18.0 ± 3.6 (0.86 ± 0.01)	23.2 ± 2.9 (1.10 ± 0.01)^**b**^	**0.004**
TSH, ng/mL	No (n=34)	1.5 ± 0.2 (1 ± 0)	0.8 ± 0.1 (0.53 ± 0.01)^**a**^	1.3 ± 0.3 (0.87 ± 0.01)	1.5 ± 0.2 (1.00 ± 0.01)^**b**^	**<0.001**
	Yes (n=47)	2.0 ± 0.3 (1 ± 0)	1.2 ± 0.4 (0.60 ± 0.01)	1.2 ± 0.1 (0.60 ± 0.01)^**a**^	2.1 ± 0.2 (1.05 ± 0.01)^**c**^	**<0.001**
PRL, ng/mL	No (n=34)	459.4 ± 147.9 (1 ± 0)	183.0 ± 67.1 (0.40 ± 0.01)^**a**^	182.2 ± 68.6 (0.40 ± 0.01)^**a**^	180.2 ± 64.5 (0.39 ± 0.01)^**a**^	**<0.001**
	Yes (n=47)	23.2 ± 3.5 (1 ± 0)	7.9 ± 1.0 (0.34 ± 0)^**a**^	8.0 ± 0.8 (0.34 ± 0)^**a**^	9.4 ± 1.0 (0.41 ± 0)^**a,b**^	**<0.001**
FSH, ng/mL	No (n=34)	4.3 ± 0.4 (1 ± 0)	3.7 ± 0.4 (0.86 ± 0.01)	3.6 ± 0.4 (0.84 ± 0.01)	4.7 ± 0.5 (1.09 ± 0.01)^**b,c**^	**0.001**
	Yes (n=47)	19.2 ± 4.2 (1 ± 0)	11.3 ± 2.1 (0.56 ± 0.01)^**a**^	11.4 ± 2.2 (0.59 ± 0.01)^**a**^	14.9 ± 3.0 (0.78 ± 0.01)^**b,c**^	**<0.001**
LH, ng/mL	No (n=34)	3.2 ± 0.5 (1 ± 0)	2.2 ± 0.4 (0.69 ± 0.01)^**a**^	2.6 ± 0.4 (0.81 ± 0.01)	4.4 ± 0.9 (1.38 ± 0.02)^**b**^	**0.006**
	Yes (n=47)	5.8 ± 1.2 (1 ± 0)	4.4 ± 1.0 (0.76 ± 0.01)^**a**^	4.4 ± 0.9 (0.76 ± 0.01)^**a**^	6.8 ± 1.2 (1.17 ± 0.01)^**b,c**^	**<0.001**
Total hormone index^2^	No (n=34)	6.0 ± 0	4.0± 0.01	4.1 ± 0.01	5.1 ± 0.01	**<0.001**
	Yes (n=47)	6.0 ± 0	3.6± 0.01	3.7 ± 0.01	5.2. ±0.01	**<0.001**

Data are presented as estimated mean ± standard error of mean (SEM) by generalized estimation equation and (mean ratio to preoperative measurement ± SEM). When a SEM of 0 is shown, it means the SEM is very small and approximates to 0.

Abbreviations: GH: growth hormone; ACTH: adrenocorticotropic hormone; TSH: thyroid-stimulating hormone; PRL: prolactin; FSH: follicle-stimulating hormone; LH: luteinizing hormone; NA: not available; NFPA: non-functioning pituitary adenomas.

^1^ Log2 or Log10 transformation was applied.

^2^ Total hormone index is equal to the sum of ratios of postoperative hormone levels to preoperative hormone levels.

a. Significant difference between the given time point and pre-operation, *P* < 0.05.

b. Significant difference between the given time point and Day 1, *P* < 0.05.

c. Significant difference between the given time point and Day 7, *P* < 0.05.

In NFPA patients, the GH level was not significantly changed over time. However, preoperative mean PRL level decreased at postoperative day 1 (from 23.2 to 7.9 ng/mL) and slightly increased to 9.4 ng/mL at 4 months follow-up. Mean ACTH, TSH, FSH and LH levels were also significantly decreased at postoperative day 1 compared to their preoperative levels (*P*<0.05), and then returned to baseline values at 4 months follow-up.

The total hormone index, which is a sum of the ratios between pre- and postoperative hormonal status of PRL, GH, FSH, LH, TSH and ACTH, was also analyzed before the surgery and during the follow-up period. As compared to preoperative values, the total hormone index decreased immediately following surgery, increasing over the follow-up period to similar extents in patients with and without NFPA (Table 2).

Analysis of only null-cell type NFPA patients revealed similar results. Although a decline was noted in ACTH, TSH, FSH and LH levels at either day 1 or day 7 follow-up, they returned to baseline levels at 4 months follow-up (Supplemental Table 1). However, after a rapid reduction at postoperative day 1, PRL levels stabilized. Similarly, the total hormone index value decreased immediately following surgery, and then increased at 4 months follow-up.

### Influence of tumor histological types on pituitary hormone levels and total hormone index

**Figure 1:**
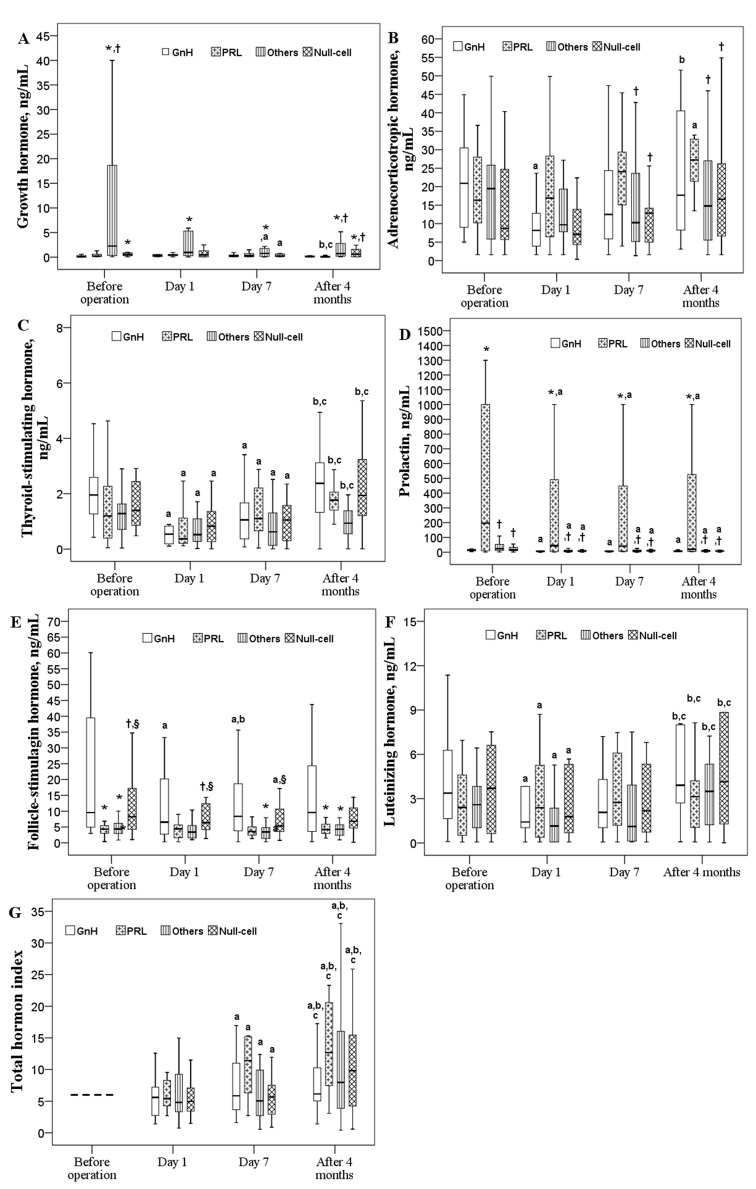
Changes in six pituitary hormones over time in patients with different histological types (n = 81) Box plot consists of median (middle line), the P^25^ (bottom of the box) and the P^75^ (top of the box) percentiles. Log transformation was preceded prior to doing analysis. Data were tested by generalized estimation equation (GEE). ^a-c^ significantly different from baseline ^a^, postoperative day 1 ^b^, or postoperative day 7 ^c^, *P* < 0.05. * significantly different from the GnH type, *P* < 0.05. † significantly different from the PRL type, *P* < 0.05. § significantly different from other types, *P* < 0.05.

**Figure 2:**
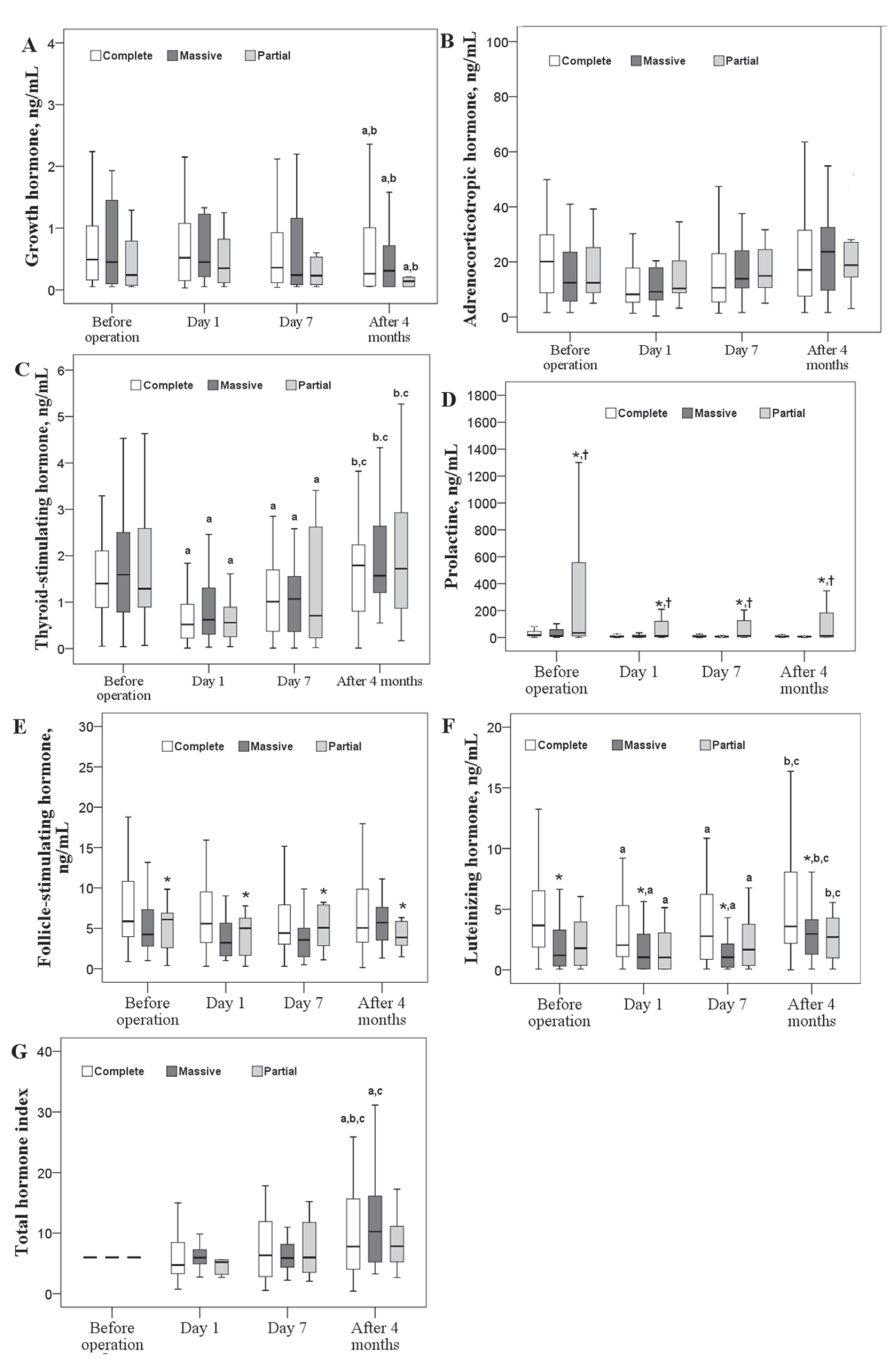
Changes in six pituitary hormones over time according to different resection types Box plot consists of median (middle line), the P^25^ (bottom of the box) and the P^75^ (top of the box) percentiles. Log transformation was preceded prior to doing analysis. Data were tested by generalized estimation equation (GEE). ^a-c^ significantly different from baseline ^a^, postoperative day 1 ^b^, or postoperative day 7 ^c^, *P* < 0.05. * significantly different from the GnH type, *P* < 0.05. † significantly different from the PRL type, *P* < 0.05. § significantly different from the other types, *P* < 0.05.

Differences in pituitary hormone levels based on tumor histological classification are illustrated in Figure 1. Significant time effects on pre- and postoperative levels of TSH, PRL, and LH as well as the total hormone index were detected (*P*<0.05). GH levels were significantly decreased at postoperative day 7 in patients with other types (TSH-, PRL-, and LH-secreting) or null-cell type of adenoma (*P*<0.05; Figure 1A). A significantly lower ACTH level was noted in patients with GnH adenoma type at postoperative day 1 although it rose to the baseline level at 4 months. In those with PRL adenoma type, ACTH levels were increased 4 months after the operation (*P*<0.05; Figure 1B). Regardless of histological adenoma type, PRL levels decreased at postoperative day 1 and remained low thereafter (Figure 1D). Low FSH levels were found at day 1 and day7 for GnH group and at day 7 for Null-cell group (Figure 2E). TSH and LH levels decreased significantly at postoperative day 1 (*P*<0.05), and then returned to their mean preoperative levels at 4 months (Figure 1C and 1F). The total hormone index slightly decreased at postoperative day 1 and then increased in all types of adenoma (*P*<0.05; Figure 1G).

Levels of GH, ACTH, PRL, and FSH were significantly different between the various histological types. Specifically, the pre- and postoperative GH levels in patients with the GnH adenoma were significantly lower through whole study period (*P*<0.05). Patients with PRL adenoma had significantly lower GH levels at baseline and at 4 months follow-up (*P*<0.05). Differences in the ACTH level between the PRL group and the null-cell group as well as the other groups were found after postoperative day 7, with higher ACTH levels found in the PRL group (*P*<0.05). In general, the pre- and postoperative FSH levels in the other groups were significantly lower than those in the GnH group (*P*<0.05). As we expected, the PRL group had the highest pre- and postoperative PRL levels. (Figure 1)

### Influence of type of tumor resection on postoperative pituitary hormone levels and total hormone index

No significant differences were observed in the levels of GH, ACTH, TSH, and the total hormone index when compared across resection types (Figure 2A-2C, 2G, respectively). However, PRL levels were significantly higher in patients with partial tumor resection (*P*<0.05; Figure 2D); FSH and LH levels were significantly higher in patients with complete tumor resection (*P*<0.05; Figure 2E and 2F, respectively).

**Figure 3:**
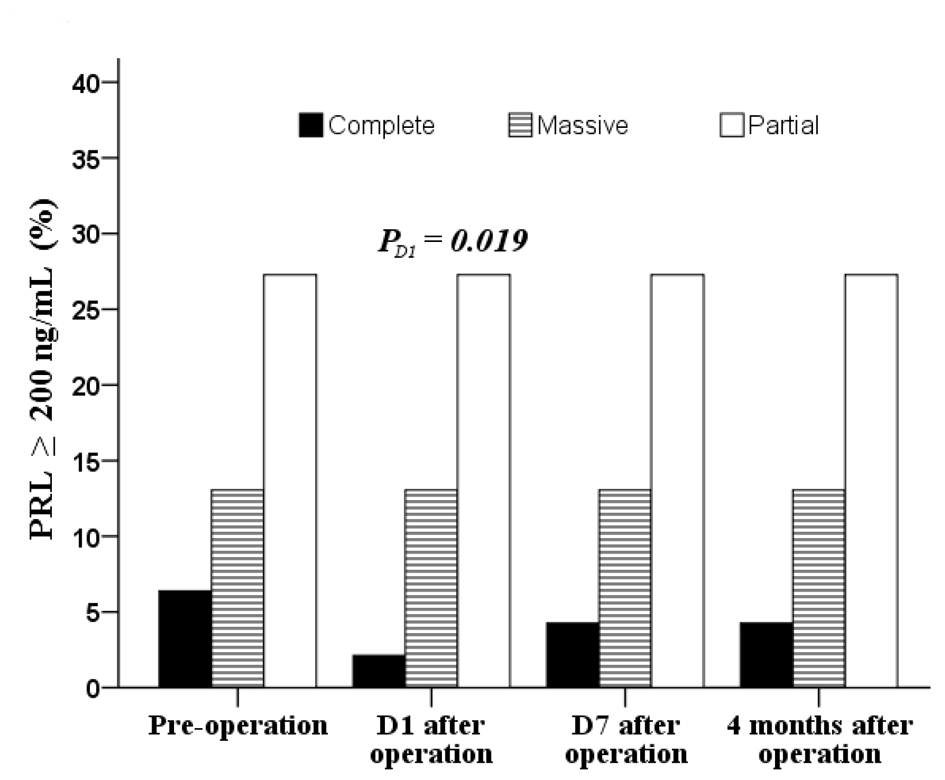
Distribution of pre- and post operative patients with PRL ≥ 200 ng/mL Data were tested by Chi-square test at baseline and Fisher’s exact test for other time points.

After the operation, GH levels were significantly decreased across the type of tumor resection while the total hormone index in the complete and massive resection groups increased gradually after a minor decease at postoperative day 1 (*P*<0.05; Figure 2A and 2G, respectively). After a significant decrease at day 1 follow-up, TSH and LH levels increased again to baseline levels (Figure 2C and 2F, respectively). At postoperative day 1, a higher percentage of patients receiving partial resection had high PRL (≥200 ng/mL) than those receiving complete resection (27.3% vs. 2.1%, *P* = 0.019; Figure 3).

### Changes in the proportion of hypopituitarism after surgery

The proportion of patients with hypopituitarism increased to 44.4% and 45.7% at postoperative days 1 and 7, respectively and then went down to 23.5% at 4 months follow-up. However, these changes did not reach statistical significance (Table 3).

**Table 3:** Pre- and postoperative hypopituitarism (n=81)

	Preoperative		
Postoperative	No hypopituitarism	Hypopituitarism	*p*
*Day 1*			0.115
No hypopituitarism	39 (73.6)	6 (21.4)	
Hypopituitarism	14 (26.4)	22 (78.6)	
*Day 7*			0.108
No hypopituitarism	36 (67.9)	8 (28.6)	
Hypopituitarism	17 (32.1)	20 (71.4)	
*Month 4*			0.078
No hypopituitarism	47 (88.7)	15 (53.6)	
Hypopituitarism	6 (11.3)	13 (46.4)	

Data are presented as count (%) and tested by McNemar test.

### Effectors of preoperative hypopituitarism

Results of univariable and multivariable logistic regression analyses are shown in Table 4. Age and gender were the only two significant factors associated with preoperative hypopituitarism. A higher risk of hypopituitarism was found in patients aged between 38-49 y (odds ratio [OR] = 4.23, 95% confidence interval [CI]: 1.06-16.82, *P* = 0.041) than in patients <38 y. Males were more likely to have pre-operative hypopituitarism (OR = 4.88, 95% CI: 1.64-14.55, *P* = 0.004) than females.

**Table 4:** Effectors of preoperative hypopituitarism revealed by logistic regression analysis

	Univariable		Multivariable	
	OR (95% CI)	*p*	OR (95% CI)	*p*
Age, years				
≤ 37	Reference		Reference	
38-49	4.93 (1.33, 18.31)	.017	4.23 (1.06, 16.82)	.041
> 49	4.31 (1.18, 15.81)	.027	3.25 (0.83, 12.74)	.091
Male gender	5.59 (1.94, 16.08)	.001	4.88 (1.64, 14.55)	.004
Type of adenoma				
Gonadotropin	Reference			
Prolactin	1.3 (0.31, 5.39)	.718		
Null-cell	1.56 (0.42, 5.85)	.510		
Others^1^	1.6 (0.41, 6.21)	.497		
NFPA adenoma	1.49 (0.58, 3.83)	.408		
Tumor size, mm	1.14 (0.66, 1.98)	.634		
Tumor volume, mm^3^	1.01 (1, 1.02)	.090		
Level of resection				
Complete	0.56 (0.15, 2.14)	.399		
Massive	0.64 (0.15, 2.77)	.550		
Partial	Reference			
Grade of cavernous sinus invasion	0.78 (0.47, 1.29)	.326		
Grade of suprasellar extension	1.07 (0.66, 1.74)	.771		
Total score of invasion	1 (0.8, 1.24)	.972		
Cystic degeneration	1.06 (0.42, 2.67)	.907		

Abbreviations: OR: odds ratio; CI: confidence interval; NFPA: non-functioning pituitary adenomas

## DISCUSSION

In the present study, we measured pituitary hormone levels individually at baseline and postoperatively in patients undergoing transsphenoidal resection of pituitary adenomas. In addition, we applied a novel total hormone index that is equivalent to the summed ratios of the postoperative levels of all pituitary hormones to preoperative levels (see the calculation in the Methods section). Our results showed that pituitary hormone levels decreased immediately after resection of both NFPAs and functional pituitary adenomas, returning to preoperative levels at 4 months follow-up. Similarly, the total hormone level of the pituitary gland decreased immediately following surgery, and then increased continuously throughout the 4-month follow-up period. We found that partial resection for non-secreting tumors also reduced hormone levels, which was likely due to blood supply to the pituitary tissue that may be affected during surgery. At 1 week after the surgery as pituitary function recovers, hormone levels began to increase slowly. Full recovery at 4 months postoperative showed a return of hormone levels to preoperative levels. Given this pattern, the total hormone index may serve as a valuable indicator of postoperative changes in pituitary function.

Pituitary adenoma resection had a considerable impact on PRL levels in the present study, which were significantly decreased on first day following surgery, which is consistent with previous studies [16, 17]. Serum PRL levels were reduced to normal in 872 of 1224 patients during the early postoperative period, increasing to preoperative levels in 21% of patients within one year after surgery [17]. The relationships between PRL levels, tumor size, and extent of resection have been previously described [18, 19]. In 123 patients with PRL-type pituitary adenomas, patients with large-volume tumors and obvious cavernous sinus invasion had correspondingly high PRL levels [19]. PRL levels do not typically normalize; the success rate of surgery is low, and the complication rate is high [20]. In the present study, PRL levels were significantly higher in patients with partial tumor resection than those in patients with massive or complete tumor resection, and FSH and LH levels were significantly higher in patients with complete tumor resection than in patients receiving massive or partial tumor resection. Although significant decreases were noted in PRL levels especially, a notably higher percentage of patients receiving partial resection had high PRL (≥200 ng/ mL) compared to patients receiving complete resection and long-term observation was required.

Because the symptoms and signs caused by the hormone deficiency can often be non-specific and vague, all patients with a pituitary lesion should be evaluated for hypopituitarism [21]. Although most transsphenoidal surgeries may induce hypopituitarism, few studies have systematically analyzed factors related to hypopituitarism. In the present study, multifactorial analysis showed that preoperative hypopituitarism was significantly associated with male sex, which is consistent with previous studies showing gender differences in tumor biology and clinical outcomes with specific pituitary adenoma subtypes. Analysis of patients with prolactinomas [22] and GH-secreting pituitary adenomas [23] revealed sex differences in that men had more aggressive disease and poorer outcomes. In contrast, NFPAs were smaller and the outcomes were better for male patients [24].

Multifactorial analysis also showed that preoperative hypopituitarism was significantly associated with age in the present study. The association between age and hypopituitarism has also been noted in previous studies [5, 25]. In patients with NFPAs, patient age was associated with postoperative functional recovery [26]. Furthermore, the likelihood of recovery following transsphenoidal adenomectomy is greater in younger patients [27]. During our lifetime, dynamic changes may occur in the size, morphology and signal intensity of the pituitary gland, reflecting changes in pituitary hormone levels. The vertical diameter of the pituitary gland increases gradually after birth and reaches maximum size in late adolescence and during lactation in women. The adenohypophysis reaches maximum size between ages 20 and 29, and then gradually shrinks along with decreased blood supply and deepened depressions during aging. It may increase again in women aged 50-59, possibly due to decreased circulating levels of cortisol and increased release of gonadotrophin in menopausal women [15]. After age 65, empty sella may even be observed in some people. Age-related changes in the pituitary gland arise from reduced volume of normal pituitary tissue and decreased stem cell function, consequences of reduced blood supply from age-related changes in microcirculation.

Although the prevalence of hypopituitarism is 45 per 100,000 individuals, many people remain undiagnosed [28] as diagnosing hypopituitarism in elderly patients is a challenge. Careful observation of progressive symptoms and differential assessment of the hormonal axes, including cortisol, TSH, free-T4, LH, FSH, sex hormones, as well as conducting ACTH stimulation testing may be required [29]. Undiagnosed and untreated hypopituitarism increases mortality [15], and replacement or restoration of pituitary hormones can improve patients’ quality of life and reduce risk of morbidity and mortality [30]. Further studies are required to determine if the total pituitary hormone index described here could identify those patients with undiagnosed pituitary dysfunction.

In an evaluation of 223 patients with silent pituitary adenomas, univariate and multivariate analysis revealed that the probability of pituitary axis dysfunction increased with larger tumor volume [31]. Essentially, enlargement of the tumor volume increases the intrasellar pressure, which decreases the amount of blood flow in the hypophyseal portal system and hypophyseal stalk, thereby decreasing the amount of hormones delivered to the pituitary from the hypothalamus. For patients with pituitary adenomas, compression of the hypophyseal portal system and hypophyseal stalk together with partial avascular necrosis of the adenohypophysis are considered to be the main mechanisms of hypopituitarism [30, 32, 33]. Microsurgical transsphenoidal surgery is shown to be safe and efficacious for pituitary adenoma resection in all patients except for those with prolactinomas that are responsive to medical therapy [34]. Pituitary surgery may improve the pituitary function of patients with hypopituitarism caused by pituitary adenoma, and pituitary function can be restored in nearly half of patients after surgery [35]. In the present study, preoperative hypopituitarism was not correlated with tumor volume, which may be due to the small sample size.

Assessment of the pituitary function is currently limited to analyzing a single hormone that is either elevated with accompanying symptoms or reduced with pituitary insufficiency and does not take into account the relationships between various hormones. Previous studies have shown evidence that the trigeminocardiac reflex (TCR) is a negative prognostic factor of pituitary function following surgery for pituitary adenomas [35]. A novel “total pituitary hormone index” that reflects changes in overall pituitary function has been proposed in this study. As the sum of various hormone changes, the total hormone index may represent a quantitative indicator, which can reflect the overall level of pituitary secretion changes after surgery because it includes hormones with normal levels before surgery and those not causing related symptoms directly, to evaluate the changes in the endocrine function after various treatments (surgery, medication, radiotherapy, etc.). Because the surgery itself can alter pituitary hormone secretion, it is possible that the levels of some hormones increase while that of some other hormones decrease within a single individual. Thus, a method to estimate the overall changes in all pituitary hormones may be representative of the gradual functional recovery of the pituitary gland following the trauma induced by the surgery itself and may guide clinical decision making regarding the initiation and continuation of individualized hormone replacement therapy. In the present study, the total hormone level decreased on the first postoperative day, gradually increasing thereafter. This indicates that the surgery damages the pituitary tissue around the tumor likely via direct mechanical injury and partial blood supply interruption, which induces a temporary decrease in the total hormone level after surgery. However, the total hormone level gradually increases with the progressive recovery of the pituitary gland. It is likely that the total hormone level will become stable following the complete recovery of the pituitary gland. Therefore, clinicians should be aware that adenohypophysis function of patients with preoperative hypopituitarism may not improve immediately after surgery but on the contrary decrease, necessitating hormone replacement therapy in some cases. The total hormone level may also decrease after surgery in patients without preoperative hypopituitarism, and we should not be misled by the elevated levels of individual hormones due to intraoperative adenohypophysis injury. However, further studies are necessary to correlate the values obtained using this index and long-term clinical outcomes to fully evaluate its clinical relevance and prognostic value.

In addition to being simple and convenient, the total hormone index had excellent repeatability and reliability, especially given that the patients received postoperative hormone replacement therapy, but not hormonal drugs, so there was little disturbance of hormone secretion. Specifically, comparisons of the preoperative hormone values tested after the patients were admitted with the values obtained from outpatient testing before admission showed that the measurements were consistent with only minor discrepancies between the hormone levels (data not shown).

The concept and method of analyzing the total hormone index is most suitable for non-secreting pituitary adenomas. Although it is not likely to be clinically applicable for secreting pituitary adenomas, this method may be useful for those tumors that secrete fragments of hormone, which have no biological activity and cannot be detected by the current chemiluminescent method. In addition to calculating overall hormone levels in non-secreting pituitary adenomas, this paper also attempted a preliminary investigation by analyzing the change values for the six types of hormones in secreting adenomas (data not shown). Because the overall hormone values for functional adenomas may not only include hormones secreted from the pituitary, but also some hormones secreted by the adenoma, the sum of the value of six types of hormones may not distinguish whether the pituitary’s functional level is high or low, but rather the overall secretion results of the “pituitary-pituitary adenoma complex” as a whole (which regards the tumor and pituitary as a single endocrine organ). This approach does not reflect the pure postoperative pituitary endocrine level, but rather the overall pituitary hormone level in the body. Regardless of whether the tumor has been eliminated, this approach can facilitate assessment of the body’s overall pituitary hormone level. It may be, therefore, a valuable approach, and has a certain reference value in determining whether to undertake postoperative hormone replacement.

In addition to its retrospective design, the present study has several limitations. We checked pituitary hormone levels individually at distinct time points, which limited the ability to distinguish between temporary and permanent hypopituitarism. The specific time of day in which samples were drawn for hormone analysis may bias the results. Additionally, we did not apply specific diagnostic tests to identify patients with hypopituitarism, and cases were exceptionally diversified so that details of the different types of adenomas were not followed. Therefore, we do not know if specific patient subsets experience greater improvements over others. We also did not have the equipment or capability to measure IGF-1 levels in all of the patients as it was introduced to our hospital in 2013, and tropin response following stimulation tests as well as antidiuretic hormone (ADH) assessments were not conducted due to logistical difficulties. Furthermore, we cannot explain the finding of higher FSH and LH values in patients with complete tumor resection compared to partial resection, and further study is needed. Also, menopause data were not included; therefore, we did not know whether female patients were pre- or postmenopausal, which could influence hormone levels. Finally, no long-term patient follow-up data were available. Thus, additional prospective studies with long-term postoperative follow-up and further exploration of the usefulness of the total hormone index are necessary to improve our understanding of hypopituitarism in patients with pituitary adenoma. Future experiments are also warranted to test the validity and generalizability of the “total pituitary hormone index.”

## CONCLUSIONS

Transsphenoidal resection of a pituitary adenoma reduced pituitary hormone levels immediately after surgery, returning to preoperative levels at 4 months after surgery. Similar time trends were found in all patients with NFPA tumors. Similarly, the “total hormone level” decreased at postoperative day 1, and then increased throughout the 4-month follow-up period. In patients with PRL pituitary adenomas, PRL levels >200 ng/mL correlated positively with the degree of pituitary adenoma resection. Age and male sex were independent factors associated with preoperative hypopituitarism.

## SUBJECTS AND METHODS

### Study design and subjects

A retrospective, single-center study was conducted, analyzing 218 consecutive patients with pituitary adenomas who underwent transsphenoidal surgery between January 2011 and December 2013 in the Neurosurgery Department of our hospital. Inclusion criteria were (1) a complete medical record and pre- and postoperative data from the study hospital (including pituitary hormone status before surgery and at 1 day, 7 days, and 4 months follow-up; pathology reports; preoperative and postoperative MRI films; surgical records and daily progress notes); (2) surgery performed by the same surgeon; (3) age of older than 18 y; and (4) a diagnosis of pituitary adenoma confirmed by immunohistochemistry. Exclusion criteria were (1) a history of prior surgery for pituitary adenoma; (2) the presence of primary endocrine disorders (primary hyperthyroidism or Cushing’s syndrome caused by adrenocortical tumor); (3) history of hormone replacement therapy 4 months before and after surgery; (4) pre-surgical radiotherapy; and (5) use of drugs that significantly alter hormone levels. A total of 81 patients met the inclusion criteria and were enrolled, including 43 males and 38 females with mean age 44.81±12.30 y (range, 19-75 y). With the exception of patients with Cushing’s disease, all other patients received one time intravenous dexamethasone (10 mg) during the intra-operative period.

Immunohistochemical classification analysis as described previously [36] showed 18 PRL adenomas, nine GH adenomas, nine multiple hormone adenomas, 18 gonadotropin cell adenomas, two TSH adenomas, and four ACTH adenomas. Diagnoses based on clinical manifestations and endocrine levels included 47 patients with NFPA (21 null cell tumors, 18 gonadotropin cell tumors, and eight silent-type cell tumors).

### Preoperative MRI examinations and evaluation

Sagittal and axial T1-weighted images, coronal and axial T2-weighted images, coronal and contrast-enhanced Flair images of all subjects were obtained using the Siemens 3.0T MAGNETOM Trio Tim MRI system (Siemens, Munich, Germany). Routine MRI scanning parameters: turbo spin echo (TSE) sequence was used for T1WI, TE = 8-15 ms, TR = 400-500 ms, three times of excitation; TSE sequence was used for T2WI, TE = 83-98 ms, TR = 3000 ms, two times of excitation. Field of view (FOV) = 180×180 mm, matrix = 320-384×240-252, slice thickness and gab of the axial scan were 5 mm and 6.5 mm, respectively; slice thickness and gab of the sagittal and coronal scans were 2.5 mm and 2.75 mm, respectively. After plain scanning, contrast-enhanced scanning was performed using Gd-DTPA (0.2 mmol/kg), using the same parameters as described above.

MRI data of all subjects within 1 week before surgery and after surgery were collected. Two neurosurgeons and one radiologist analyzed these data together and agreed on the definition of tumor invasiveness and cystic changes. The main observation included (1) measurement of tumor volume and preoperative maximum tumor diameter; (2) determination of cystic changes in the tumor; and (3) evaluation of tumor invasiveness.

### Calculation of tumor volume

Based on the Cavalieri principle used in stereology [37, 38], namely systematic sampling, several isometric random parallel sections crossing the target were made in either direction, the total area of all sections was multiplied by the distance between sections, and the calculated result was taken as the unbiased estimation of the volume of the target. In order to reduce the error caused by the impact of the cavernous sinus on the tumor volume after contrast-enhanced scanning, we defined the part between every two planes on the coronal plane of the tumor as an independent frustum. The authors’ previously published [39] formula for calculating the frustum volume is: V = [S1 + S2 + (S1 + S2)/2] × h/3 = (S1 + S2) × h/2 (S1 and S2 were measured by the INFINITT PACS system). A sample calculation of the tumor area on a single coronary MRI plane is shown in supplemental Figure 1.

### Measurement of the tumor diameter

Pituitary adenomas were divided into microadenoma, macroadenoma and giant adenoma, as previously described [40]. A pituitary adenoma <10 mm in diameter was defined as microadenoma, ≥10 mm but <40 mm was defined as macroadenoma, and ≥40 mm was defined as giant adenoma. Among all tumors, three were microadenoma (diameter <10 mm), 72 were macroadenoma (diameter ≥10 mm and <40 mm) and six were giant adenoma (diameter ≥40 mm), based on tumor diameters as previously described [27]. The maximum diameter of the tumor on the coronal plane was measured using the INFINITT PACS system as shown in supplemental Figure 2.

### Evaluation of the extent of tumor invasiveness

No unified standard has been established for evaluating the invasiveness of pituitary adenomas. The relationship between the pituitary adenoma and its peripheral tissues was evaluated using a method described by Sarlis et al. [41]. It involved three aspects, such as cavernous sinus invasion, sphenoid sinus invasion and suprasellar extension (Table 1). Each item was scored individually. The total scores of extent of invasiveness = scores of cavernous sinus invasion + scores of sphenoid sinus invasion + scores of suprasellar extension.

### Hormone level measurements

Bloods samples were collected from the at-rest cubital vein of all patients two days prior to surgery at 8:00 a.m. in quiet conditions, and again on postoperative days 1 and 7 and at 4 months at the same time of day and under the same conditions. All hormone levels were measured, including GH, ACTH, FT3, FT4, E2, T, TSH, PRL, FSH, LH, and cortisol. Complete follow-up hormone measurements were performed at or after 4 months postoperatively. All hormones tests were done by chemiluminescence detection methods using the Siemens ADVIA Centaur XP machine (Siemens, Munich, Germany). The reference values for each hormone are as follows: GH, <5 ng/mL; ACTH, 4.7-48.8 pg/mL; T3, 0.92-2.79 ng/mL; T4, 58.1-140.6 ng/mL; FT3, 3.5-6.5 pmol/L; FT4, 11.5-22.7 pmol/L; TSH, 0.35-5.5 μIU/mL; PRL, not pregnant 2.8-29.2 ng/mL, menopausal 1.8-20.3 ng/mL, ovulating 3.4-33.4 ng/mL; FSH, pregnancy 9.7-208 mIU/mL, follicular phase 2.5-10.2 mIU/mL, the corpus luteum of 1.5-9.1 mIU/mL; and LH, follicular phase 1.9-12.5 mIU/mL and luteal phase 0.5-16.9 mIU/mL. Hypopituitarism was defined as a deficiency of one or more pituitary hormones [14].

### Total hormone index calculation

In order to reflect the overall secretion of pituitary hormones, we have proposed the concept of a “total hormone index” measuring the six pituitary hormones by group. The ratios between pre- and postoperative hormonal status of each hormone (i.e., PRL, GH, FSH, LH, TSH and ACTH) were calculated, then the ratios were added together to obtain the “total hormone index.” The ratio of the postoperative hormone level to the preoperative hormone level was taken as the change rate, which allowed comparison of the various postoperative hormone levels to the preoperative hormone levels. Each preoperative hormone level was taken as “1” unit, and the sum of ratios of postoperative hormone levels to corresponding preoperative levels was described as the “total hormone index.” For example, the total hormone index on the first postoperative day is equal to postoperative first day GH level/preoperative GH level + postoperative first day ACTH level/preoperative ACTH level + postoperative first day TSH level/preoperative TSH level + postoperative first day PRL level/preoperative PRL level + postoperative first day FSH level/preoperative FSH level + postoperative first day LH level/preoperative LH level. The preoperative “total hormone index” was recorded as 6.

### Criteria for tumor resection and tumor invasiveness

The criteria for tumor resection, which were modified by Hoffman classification [42], are (1) total/complete resection; (2) massive resection (i.e., removing >60% of the tumor); and (3) partial resection (i.e., removing <60% of the tumor). The tumor invasiveness criteria included grades 0, 1, 2, and 3, as previously described [40]. Pituitary dysfunction was defined as having at least one of three hormone-related dysfunctions as follows: gonadal axis, thyroid axis, adrenal axis dysfunction, as previously described [42].

### Microscopic endonasal surgical procedure

All surgeries were performed by a single surgeon, first author, Dr. Wang, who has more than 25 years’ experience with the microscopic endonasal surgical procedure. This procedure is microscopic and not endonasal endoscopic surgery. Tumor resection was performed under general anesthesia. The patient was placed in the supine position with 15-20° of neck extension. The right-sided nostril-nasal septum-transsphenoidal approach was used in all operations, as previously described [39]. A straight incision was made in the middle of the nasal septum (3 cm posterior to the anterior nostril). The anterior wall of the sphenoid sinus was reached between the right mucoperiosteum and the perpendicular plate of the ethmoid bone. After the sellar floor was reached by opening the anterior wall of the sphenoid sinus and removing the bone of sella turcica, the dura mater was cut open in an “X” shape. The tumor was removed in pieces by using ring curettes, tumor-grasping forceps, and suction devices. The posterior part of the tumor was removed first, bilateral sides were then removed, and the anterosuperior part was removed last. A gelatin sponge was used to fill the tumor cavity that was left after collapse of the sellar diaphragm. After surgery, the bilateral nasal cavities were filled with Vaseline Gauze. The tumor tissue specimen was sent to the pathology department of our hospital to confirm the immuno- histochemistry-based tumor classification.

### Pathology examination of tumor tissue

All surgical specimens were prepared in the pathology department and examined by the pathologist. The tumor tissues were fixed in 10% neutral buffered formalin solution, embedded in paraffin, sectioned, and stained with HE, PRL, GH, ACTH, TSH, FSH, and LH, which were all purchased from DAKO, Agilent Technologies Company (Carpinteria, CA, USA) and used as 1:500 dilutions for all primary antibodies. Under bright field microscopic observation, fields with >10% of stained cells were defined as positive. The final classification followed the WHO 2004 classification criteria [43].

### Statistical analysis

Continuous variables are presented as mean ± standard deviation (SD). Categorical variables are expressed by count (%) and tested by the chi-square test; however, since the chi-square test is not reliable for tables with expected cell frequencies of less than 5, Fisher’s exact test was used instead if 20% of the cells had expected values of <5, as previously described [44]. The McNemar test was used for categorical data to test changes in the proportion of hypopituitarism after surgery. The effects of time, tumor resection type, and histological type on six pituitary hormones (i.e. GH, ACTH, TSH, PRL, FSH, and LH) and the total hormone index of those six hormones were examined by the generalized estimation equation (GEE), which is characterized by its unbiased estimations of regression parameters even for variables that are not normally distributed [45]. Once a significant effect of time or histological type was revealed, Bonferroni’s correction was performed for pairwise comparisons. Interaction terms comprising time and either the tumor resection type or the histological type were included in the models. If a significant interaction term was revealed, strata analyses by the resection type or the histological type were performed. Log10 or Log2 transformation was applied before proceeding GEE. Logistic regression analysis was performed to determine factors of preoperative hypopituitarism. Significant variables revealed in the univariable analysis were used for subsequent multivariable analysis. All statistical assessments were evaluated at a two-sided alpha level of 0.05 using IBM SPSS software, version 22 (IBM Corp., Armonk, NY, USA).

## SUPPLEMENTARY MATERIALS FIGURES AND TABLE


