# Supplementary material for: A novel “total pituitary hormone index” as an indicator of postoperative pituitary function in patients undergoing resection of pituitary adenomas
Source: Oncotarget. 2017 Mar 7;8(45):79111–25. doi: 10.18632/oncotarget.15978 (PMC5668024; doi:10.18632/oncotarget.15978)
Supplement: Supplementary file 1 [file oncotarget-08-79111-s001.pdf]

A novel "total pituitary hormone index" as an indicator of postoperative pituitary function in patients undergoing resection of pituitary adenomas

Supplementary Material

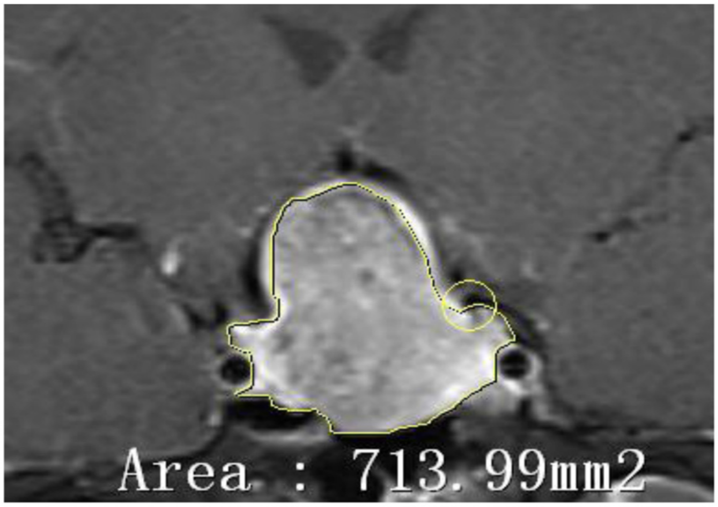

Supplemental Figure 1. Calculation of the tumor area on a single coronary MRI plane. The contouring region in the figure is the tumor region with area: 713.99 mm^2.

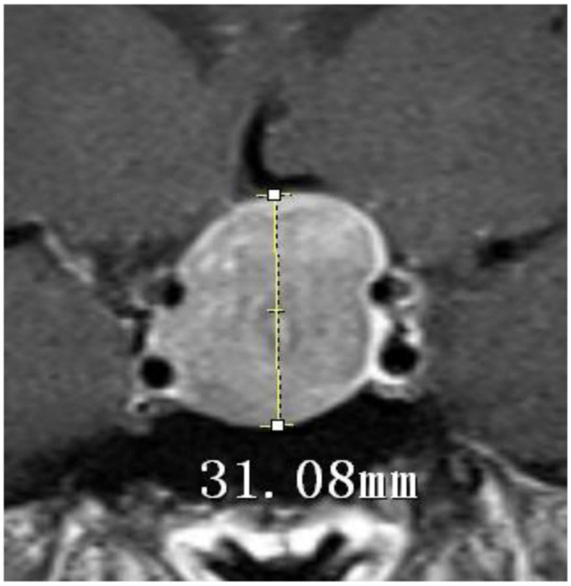

Supplementary Figure 2. Measurement of the maximum tumor diameter on a single coronal MRI plane.

Supplemental Table 1. Differences between pre- and postoperative pituitary hormone levels in 21 null-cell type NFPA patients[1]

Hormone	Preoperative	Postoperative Day 1	Postoperative Day 7	Postoperative Month 4	*p*
GH, ng/mL	1.8 ± 0.8 (1 ± 0)	0.8 ± 0.2 (0.44 ± 0.01)	0.9 ± 0.3 (0.50 ± 0.01)	1.2 ± 0.4 (0.67 ± 0.01)	**0.021**
ACTH, ng/mL	20.6 ± 5.4 (1 ± 0)	14.7 ± 5.7 (0.71 ± 0.01)	11.0 ± 1.5 (0.53 ± 0.01)	20.1 ± 3.9 (0.98 ± 0.02)	**0.035**
TSH, ng/mL	2.1 ± 0.4 (1 ± 0)	1.7 ± 0.7 (0.81 ± 0.02)	1.1 ± 0.2 (0.52 ± 0.01)	2.2 ± 0.3 (1.05 ± 0.02)[b]	**<0.001**
PRL, ng/mL	26.4 ± 5.8 (1 ± 0)	9.4 ± 1.6 (0.36 ± 0.01)[a]	9.6 ± 1.2 (0.36 ± 0.01)[a]	9.5 ± 1.4 (0.36 ± 0.01)[a]	**<0.001**
FSH, ng/mL	15.9 ± 4.6 (1 ± 0)	12.1 ± 3.2 (0.76 ± 0.02)	11.7 ± 3.3 (0.74 ± 0.02)[a]	16.5 ± 4.9 (1.04 ± 0.02)	**0.011**
LH, ng/mL	7.2 ± 2.3 (1 ± 0)	5.5 ± 1.9 (0.76 ± 0.02)	5.4 ± 1.8 (0.76 ± 0.02)	8.9 ± 2.4 (1.24 ± 0.03)[b]	**0.003**
Total hormone index[2]	6 ± 0	3.8 ± 0.02	3.4 ± 0.02	5.3 ± 0.02	**<0.001**

Data are presented as estimated mean ± standard error of mean (SEM) by generalized estimation equation and (mean ratio to preoperative measurement ± SEM).

GH: growth hormone; ACTH: adrenocorticotropic hormone; TSH: thyroid-stimulating hormone; PRL: prolactin; FSH: follicle-stimulating hormone; LH: luteinizing hormone; NA: not available; NFPA: non-functioning pituitary adenomas

[1] Log2 or Log10 transformation was applied.

[2] Total hormone index is equal to the sum of ratios of postoperative hormone levels to preoperative hormone levels.

a. Significant difference between the given time point and pre-operation, $P < 0.05$.

b. Significant difference between the given time point and Day 7, $P < 0.05$.
